# Characterization of high risk human papilloma virus genotypes associated with oropharyngeal cancers in a Nigerian population

**DOI:** 10.11604/pamj.2021.38.40.27309

**Published:** 2021-01-15

**Authors:** Benjamin Idemudia Akhiwu, Helen Oluwadamilola Akhiwu, Tolulope Afolaranmi, Nyam Chuwang, Ambrose Elugbe, Acheng Shedrach, Pam Luka, Patricia Odumosu, Patrick Oladele Olorunfemi, Samuel Agida Adoga, Olugbenga Silas, Benjamin Tagbo Ugwu, Akinola Ladeinde, Godwin Eremwan Imade, Atiene Solomon Sagay

**Affiliations:** 1 Oral and Maxillofacial Surgery Department, University of Jos, Jos University Teaching Hospital, Lamingo Permanent Site, Jos, Plateau State, Nigeria,; 2 Department of Pediatrics, Jos University Teaching Hospital, Lamingo Permanent Site, Jos, Plateau State, Nigeria,; 3 Department of Community Medicine, University of Jos, Jos University Teaching Hospital, Jos, Plateau State, Nigeria,; 4 STAMINA Genomics Laboratory, Department of Human Anatomy, Faculty of Basic Medical sciences, University of Jos, Jos, Plateau State, Nigeria,; 5 Department of Dental and Maxillofacial Surgery, Jos University Teaching Hospital, Jos, Plateau State, Nigeria,; 6 Genomics and Postgraduate Research Laboratory, College of Health Sciences, University of Jos, Jos, Plateau State, Nigeria,; 7 Biotechnology Center, National Veterinary Research Institute, Vom, Jos, Plateau State, Nigeria,; 8 Department of Pharmaceutical and Medicinal Chemistry, Faculty of Pharmaceutical Sciences, University of Jos, Jos, Plateau State, Nigeria,; 9 Department of Pharm, Microbiology and Biotechnology, University of Jos, Jos, Plateau State, Nigeria,; 10 Department of Ear, Nose and Throat surgery, University of Jos, Jos University Teaching Hospital, Lamingo Permanent Site, Jos, Plateau State, Nigeria,; 11 Department of Pathology, University of Jos, Jos University Teaching Hospital, Lamingo Permanent Site, Jos, Plateau State, Nigeria,; 12 Department of Surgery, University of Jos, Jos University Teaching Hospital, Lamingo Permanent Site, Jos, Plateau State, Nigeria,; 13 Oral and Maxillofacial Surgery Department, College of Medicine, University of Lagos, Lagos State, Nigeria,; 14 Department of OBGYN, Faculty of Clinical Sciences, College of Health Sciences, University of Jos, Jos, Plateau State, Nigeria

**Keywords:** Human papilloma virus, Nigeria, oropharyngeal carcinoma

## Abstract

**Introduction:**

head and neck cancers have essentially been a disease of the elderly but recent studies are beginning to demonstrate their increasing incidence in young people with infections such as human papilloma virus (HPV). This study was carried out to determine the prevalence of high risk Human papilloma virus (hrHPV) related oropharyngeal carcinoma and its prevalent genotypes as well as their strength of association with HIV in adult Nigerian subjects.

**Methods:**

this was a cross-sectional study of 41 patients with oropharyngeal carcinomas seen over a 2-year period. Patients had incisional and/or excisional biopsy done under anesthesia. A portion of the specimen from which the DNA was extracted was placed in Digene HC2 DNA collection device while the 2^nd^ portion for histopathological analysis was fixed using 10% Neutral Buffered Formalin (NBF) and embedded in paraffin blocks. Oropharyngeal cancer HPV genotyping was done using HPV genotypes 14 real-tm quant kit (SACACE, Italy). The data was analyzed using SPSS version 23.

**Results:**

prevalence of HPV was 17.1% with a male to female ratio of 2.7: 1. The identified genotypes were 16, 33, 35 and 52 with 28.6% of patients having more than one genotype. Most of the age groups studied were affected. Squamous cell carcinoma and ameloblastic carcinoma were the cancers associated with HPV. HPV was not identified in the HIV positive patients.

**Conclusion:**

high-risk human papilloma virus genotypes 16, 33, 35 and 52 are associated with oropharyngeal carcinoma in Nigeria but were not found in HIV patients. This finding provides a strong evidence for the use of the 9-valent prophylactic vaccine for the prevention of oropharyngeal cancer in Nigeria. Public awareness and HPV prevention strategies should reduce significantly the incidence of oropharyngeal carcinomas in our environment.

## Introduction

The term head and neck cancer (HNC) typically refers to cancers of the oral cavity, oropharynx, nasopharynx, hypopharynx, and larynx [1]. These cancers have become a significant public health problem globally with cancers of the head and neck being reported to be responsible 350,000 deaths worldwide each year [1]. It has essentially been a disease of the elderly; more than 95% occurring in persons older than 40 years of age in most regions of the world. It has also been associated with predisposing factors such as tobacco and chronic alcohol use, ingestion of smoked fish, infections especially by viruses, dietary deficiencies and industrial pollution [1, 2]. Recent studies in developed countries are however beginning to demonstrate the increasing incidence of oral squamous cell carcinoma in young people especially those who do not smoke or use alcohol [3]. This has indicated a possible etiologic role of infections such as human papilloma virus (HPV); a fact that has also been buttressed by the fact that HPV positivity was found chiefly in young oral cancer patients who consume less alcohol and tobacco [3].

HPV are a group of over 150 related viruses, 40 of which can be easily spread through direct contact from the skin and mucous membranes of infected individuals during sexual intercourse [4, 5]. Although, HPV genomic sequences have been identified in head and neck squamous cell carcinoma, a wide range of viral detection rate of between 0 - 100% have been reported. This great disparity in prevalence rate has been attributed to ethno-geographical differences, site of lesions studied, the differences in specimen type and HPV detection methods [1, 6]. There is however, still a dearth of local information and a gap in knowledge about the role of HPV and the genotypes associated with oropharyngeal cancers in Nigeria. This study was carried out to determine the prevalence of high risk Human papilloma virus (hrHPV) related oropharyngeal carcinoma and its prevalent genotypes as well as their strength of association with HIV in adult Nigerian subjects.

## Methods

The study was a descriptive cross-sectional study of patients with oropharyngeal carcinomas seen at Jos University Teaching Hospital in North-central Nigeria from May 2017 to April 2019. The following formula [7] was used to determine the sample size:

$$\text{N=}{\left(\text{Z}/\text{E}\right)}^{2}\text{P}\left(1-\text{P}\right)$$

Where N = Sample size, Z= Desired confidence level (1.96 at 96% confidence interval) P= Prevalence (using a previous prevalence of 9.4% [1] = 0.09), E = Maximum tolerable sample error = 0.10. Therefore N= (1.96 ÷ 0.10)2 0.09(1-0.09) = 31 patients; adding 10% for dropout = 34 patients. Eventually 41 patients were recruited for the study.

Ethical approval was obtained from the Institutional Review Board of the Teaching Hospital (Ethical approval number JUTH/DCS/ADM/127/XXV/49) as well as written informed consent from each study participant.

**Inclusion criteria:** patients aged 18 years and above with suspected oropharyngeal cancers.

**Patient enrollment:** all consecutive patients with suspected oropharyngeal cancers seen at the Dental and Maxillofacial Surgery Clinic, Surgical outpatient Department, Accident and Emergency and the Department of Otorhinolaryngology of Jos University Teaching Hospital who consented to participate in the study were recruited until the target was obtained. If the histological diagnosis turned out not to be oropharyngeal cancer, that patient was removed from the study and the next eligible participant was then enrolled. Information was obtained from the patients through the medium of a pretested interviewer administered questionnaire.

**Collection of tumor samples:** patients with suspected oropharyngeal carcinoma had incisional and/or excisional biopsy done under anesthesia in the maxillofacial surgery and the otorhinolaryngology units in line with the hospital protocols for such. Each fresh tissue samples from every patient was divided into two. One portion for deoxyribonucleic acid (DNA) analysis was placed in Digene HC2 DNA collection device while the second portion for histopathologic analysis was fixed using 10% Neutral Buffered Formalin (NBF) and embedded in paraffin blocks.

### Procedure for oropharyngeal cancer HPV genotyping using HPV genotypes 14 real-tm quant kit (SACACE)

**DNA extraction:** the DNA was extracted using a commercially available kit known as the QIAamp DNA Mini Kit (Qiagen, Hilden, Germany). The extraction was performed following the manufacturer´s protocol. The extracted DNA was quantified using Qubit dsDNA HS Assay Kit in a Qubit 4 Fluorometer (Thermofisher, Scientific, USA). The DNA extract was stored at -20°C until amplification.

**Detection of hrHPV genotypes through real-time Polymerase chain reaction (PCR):** amplification was done using the HPV Genotypes 14 Real-TM Quant kit (Sacace Biotechnologies®, Italy). This is a real-time multiplex PCR test for the detection of 14 hrHPV genotypes (HPV- 16; 18; 31; 33; 35; 39; 45; 51; 52; 56; 58; 59; 66 and 68) in 4 tubes and each tube contained primers of the E6 and E7 target regions of three or four types of hrHPV and human beta-globin gene as Internal Control (IC). Each DNA sample was normalized to 3ng/ µl and 10 µL of the extracted DNA was added to 15 µL of the reaction mix solution in the 4 tubes making a total reaction volume of 25 µL in each tube. Both the negative control and standards received the same master mix. The amplification was performed in a PikoReal Real-Time PCR System (Thermofisher, Scientific, USA) under the following program: 1 cycle of 95°C for 15 min; 5 cycles of 95°C for 5 seconds followed by 60 °C for 20 seconds and 72°C for 15 seconds; and finally, 40 cycles of 95°C for 5 seconds followed by 60°C for 30 seconds and 72°C for 15 seconds. The PCR is valid if the standards show signal for all the FAM, JOE, ROX, and Cy5 fluorochromes and if the negative control has no signal. The results were interpreted using the Microsoft Excel program named “HPV Genotypes 14 Real-TM.xls” (Sacace Biotechnologies®, Italy) provided by the manufacturer.

**Serology for HIV:** all patients were tested for HIV using serology testing. The serial antibody testing method was used. The rapid test kits used were the Determine (sensitivity of 100% and specificity of 97.9%) and Uni-GoldTM (sensitivity of 99.8% and specificity of 99.9%). The patients that tested positive to both tests were diagnosed as HIV positive.

**Data analysis:** the data obtained were pooled from the questionnaire designed for the study. The independent variables included socio-demographic and clinical variables like age, sex, marital status, education level, occupation, HIV status, practice of oral sex as well as tobacco smoking and alcohol use. Tobacco smoking was further divided using the center for disease control definition into never smoked and current smokers [8]. The current smokers were individuals who have smoked at least 100 cigarettes in their lifetime and are still smoking. Alcohol intake was also further divided into current use and no history of alcohol use.

The prevalence of hrHPV was determined and presented using frequencies and percentages. The various genotypes identified were documented using percentages and the association between the various genotypes and the patient´s characteristics was determined using Chi -squared test and Fisher exact test was used as a test of correction of continuity where assumptions upholding the use of chi square test were not fulfilled. The association between Tobacco use, alcohol consumption, cancer type and HPV status were also determined using fisher´s exact test, odds ratio and 95% confidence interval.

The various histological types of oropharyngeal cancers were also determined and presented in simple percentages. The age and sex distribution as well as the distribution of the various serotypes within the different histological types of cancer identified were also tabulated. The prevalence of HIV in the study participants was determined and presented in simple percentages while the association of HIV with HPV was determined using Fisher´s exact test. All the analyses were carried out using the IBM SPSS Statistics for Windows, version 23 (IBM Corp., Armonk, N.Y., USA) and p-values of < 0.05 was considered to be statistically significant.

## Results

A total of 41 patients with oropharyngeal carcinoma were recruited in this study. They were made up of 30 (73.2%) males and 11 (26.8%) females with a male to female ratio of 2.7: 1. Majority (39%) of the patients were in the 61-70 years age group; more than 60% of the study participants had only secondary level of education. Commonest presenting complaints were sore throat and dysphagia with hoarseness and cervical lymphadenopathy as the commonest clinical finding. Other socio-demographic and clinical characteristics are presented in Table 1.

**Table 1:** socio-demographic and clinical characteristics of the study population (N=41)

Variables	Frequency (percentage)	Mean (SD)
**Sex**		
Males	30 (73.2)	
Females	11 (26.8)	
**Age group (years**)		61.6 (11.0)
21-30	1 (2.4)	
31-40	0 (0)	
41-50	4 (9.8)	
51-60	13 (31.7)	
61-70	16 (39.0)	
>70	7 (17.1)	
**Occupation**		
Artisan	4 (9.8)	
Trader	9 (21.9))	
Driver	4 (9.8)	
Unemployed	18 (43.9)	
Farmer	6(14.6)	
**Education**		
Primary	5 (12.2)	
Secondary	28(68.3)	
Tertiary	8 (19.5)	
**Marital status**		
Married	24 (58.5)	
Divorced	4 (9.8)	
Widowed	13 (31.7)	
**Presenting complaint**		
Sore throat	7 (17.1)	
Dysphagia	8 (19.5)	
Hoarseness	5 (12.2)	
Jaw swelling	9 (22.0)	
Sore throat + dysphagia	12 (29.3)	
**Significant clinical findings**		
Cervical Lymphadenopathy	3 (7.3)	
Cervical Lymphadenopathy with splenomegaly	2 (4.9)	
Jaw swelling	6 (14.6)	
Hoarseness + cervical Lymphadenopathy	16 (39.0)	
Intraoral swelling	14 (34.1)	
**Tumour site**		
Tongue	2 (4.9)	
Floor of the mouth	3 (7.3)	
Mandible	5 (12.2)	
Oropharynx	21 (51.2)	
Nasopharynx	4 (9.8)	
Palate	6 (14.6)	
**Current history of smoking**		
No	21 (51.2)	
Yes	20 (48.8)	
**Current history of alcohol use**		
No	34 (82.9)	
Yes	7 (17.1)	

The prevalence of HPV was 17.1% (7/41) in the study population; 3 (27.3%) females were HPV positive while 4 (13.3%) males were HPV positive. The age distribution, identified genotypes and the sex distribution of the genotypes are all presented in Table 2. Among the patients that were HPV positive, jaw swelling and sore throat were the commonest presenting complaints while hoarseness and cervical lymphadenopathy were the commonest clinical findings. Other clinical characteristics of the various genotypes are presented in Table 3.

**Table 2:** prevalence of HPV and HPV genotypes identified among the study population

Variable		Frequency (Percentage)	
**Presence of HPV (N=41)**			
No		34 (82.9)	
Yes		7 (17.1)	
**Sex distribution of HPV (N=7)**			
Males		4 (57.1)	
Females		3 (42.9)	
**Prevalence of HPV as aggregated by sex**			
Males		4/30 (13.3)	
Females		3/11 (27.3)	
**Age distribution of HPV (N=7)**			
21-30		1 (14.3)	
31-40		0 (0)	
41-50		1 (14.3)	
51-60		2 (28.6)	
61-70		1 (14.3)	
>70		2 (28.6)	
**HPV genotypes identified**			
16		3 (42.9)	
33		1 (14.3)	
52		1 (14.3)	
16,33		1 (14.3)	
33,35		1 (14.3)	
**Sex distribution of the genotypes identified**			
**Genotypes**	**Males**		**Females**
	**n (%)**		**n (%)**
16	2 (50.0)		1 (33.3)
33	1 (25.0)		0 (0)
52	1 (25.0)		0 (0)
16,33	0 (0)		1 (33.3)
33,35	0 (0)		1 (33.3)

**Table 3:** patient characteristics versus genotypes

Genotypes	Characteristics
	**Presenting complaints (Frequency)**
16	Jaw swelling (2), sore throat with dysphagia (1)
33	Jaw swelling (1)
52	Sore throat (1)
16,33	dysphagia (1)
33,35	Jaw swelling (1)
	**Clinical findings (Frequency)**
16	Jaw swelling (1), Hoarseness with cervical lymphadenopathy (2)
33	Jaw swelling (1)
52	Hoarseness with cervical lymphadenopathy (1)
16,33	Hoarseness with cervical lymphadenopathy (1)
33,35	Jaw swelling (1)
	**Tumour site (Frequency)**
16	Mandible (1), Oropharynx (1), Nasopharynx (1)
33	Mandible (1)
52	Oropharynx (1)
16,33	Oropharynx (1)
33,35	Mandible (1)

No significant association was found between tobacco use, alcohol consumption and the presence of HPV (P= 0.41 and 1.00 respectively). There was also no significant association between the type of cancer and the presence of one or more HPV genotypes. (p=1.00). Patients with ameloblastic carcinoma were 33.3% more likely to have more than one HPV gerotype than patients with Squamous cell carcinoma as shown in Table 4.

**Table 4:** association between tobacco use, alcohol consumption, cancer type and HPV status

Variable	Presence of HPV		Total	P	odds ratio	95% CI
	Yes	No				
	n (%)	n (%)				
**Tobacco use**				* 0.41	0.356	0.06- 2.09
Yes	2 (4.9)	18 (43.9)	20(48.8)			
No	5 (12.2)	16 (39.0)	21(51.2)			
Total	7 (17.1)	34 (82.9)	41(100)			
**Alcohol consumption**				* 1.00	0.778	0.08- 7.71
Yes	1 (2.4)	6 (14.6)	7(17.1)			
No	6 (14.6)	28 (68.3)	34(82.9)			
Total	7 (17.1)	34 (82.9)	41(100)			
**Cancer type**	**HPV genotype**					
	**One genotype**	**> One genotype**				
Squamous cell carcinoma	3(42.9)	1(14.3)	4(57.1)	*1.00	0.667	0.25-18.06
Ameloblastic carcinoma	2(28.6)	1(14.3)	3(42.9)			
Total	5(71.4)	2(28.6)	7(100)			

* =Fishers exact

Squamous cell carcinoma was the commonest type of cancer seen in 31 (75.6%) patients with oropharyngeal cancer. Squamous cell carcinoma was seen in patients from age 41 and above while one of the patients with ameloblastic carcinoma was in the 21-30-year age range. The female patients studied presented with only squamous cell carcinoma, extra nodal Non-Hodgkin´s lymphoma and ameloblastic carcinoma while seven different histological types were observed in the males. It was also observed that only patients with squamous cell carcinoma and ameloblastic carcinoma had human papilloma virus. Other age, sex, and HPV distribution among the histological types of cancer seen are presented in Table 5.

**Table 5:** histological types of the oropharyngeal cancers versus age, sex and HPV genotypes

Histological type	Frequency (percentage)							
Kaposi sarcoma	1 (2.4)							
Nasopharyngeal carcinoma	1 (2.4)							
Squamous cell carcinoma	31 (75.6)							
Extra nodal Non-Hodgkin´s Lymphoma	2 (4.9)							
Adenoid cystic carcinoma	2 (4.9)							
Malignant melanoma	1 (2.4)							
Ameloblastic carcinoma	3 (7.3)							
Total	41 (100.0)							
**Histological type**	**Age group**							
	**21-30**	**41-50**	**51-60**	**61-70**			**>70**	**Total**
Kaposi sarcoma	0	1	0	0			0	1
Nasopharyngeal carcinoma	0	0	0	0			1	1
Squamous cell carcinoma	0	3	11	15			2	31
Extra nodal Non-Hodgkin´s Lymphoma	0	0	1	1			0	2
Adenoid cystic carcinoma	0	0	0	0			2	2
Malignant melanoma	0	0	1	0			0	1
Ameloblastic carcinoma	1	0	0	0			2	3
Total	1	4	13	16			7	41
**Histological type**	**Sex**							
	**Male**		**Female**			**Total**		
Kaposi sarcoma	1		0			1		
Nasopharyngeal carcinoma	1		0			1		
Squamous cell carcinoma	22		9			31		
Extra nodal Non-Hodgkin´s Lymphoma	1		1			2		
Adenoid cystic carcinoma	2		0			2		
Malignant melanoma	1		0			1		
Ameloblastic carcinoma	2		1			3		
Total	30		11			41		
**Histological types**	**HPV genotypes**							
	**16**	**33**	**52**	**16,33**	**33,35**		**Not applicable**	**Total**
Kaposi sarcoma	0	0	0	0	0		1	1
Nasopharyngeal carcinoma	0	0	0	0	0		1	1
Squamous cell carcinoma	2	0	1	1	0		27	31
Extra-nodal Non-Hodgkin´s Lymphoma	0	0	0	0	0		2	2
Adenoid cystic carcinoma	0	0	0	0	0		2	2
Malignant melanoma	0	0	0	0	0		1	1
Ameloblastic carcinoma	1	1	0	0	1		0	3
Total	3	1	1	1	1		34	41

* Not applicable are those patients with oropharyngeal cancers but are HPV negative

In this study, 2 (4.9%) of the study participants were HIV positive and there was no significant association between HPV and HIV identified in this study, as the p-value was 1.00. However, Kaposi sarcoma and extra nodal non-Hodgkin´s lymphoma were the cancer types seen in the HIV positive patients as shown in Table 6.

**Table 6:** HIV versus HPV and cancer type

Presence of HIV		Frequency (percentage)			
No		39 (95.1)			
Yes		2 (4.9)			
Total		41 (100)			
**Presence of HIV**					
	**Yes**		**No**	**Total**	**p**
	**n (%)**		**n (%)**	**N (%)**	
**Presence of HPV**					*1.000
Yes	0 (0)		7 (17.1)	7 (17.1)	
No	2 (4.9)		32 (78.0)	34 (82.9)	
Total	2 (4.9)		39 (95.1)	41 (100)	
**Types of cancer**			**Presence of HIV**		**Total**
			**Yes**	**No**	
			**n (%)**	**n (%)**	**n (%)**
Kaposi sarcoma			1 (2.4)	0 (0)	1 (2.4)
Nasopharyngeal carcinoma			0 (0)	1 (2.4)	1 (2.4)
Squamous cell carcinoma			0 (0)	31 (75.6)	31 (75.6)
Extra nodal Non-Hodgkin´s Lymphoma			1 (2.4)	1 (2.4)	2 (4.9)
Adenoid cystic carcinoma			0 (0)	2 (4.9)	2 (4.9)
Malignant melanoma			0 (0)	1 (2.4)	1 (2.4)
Ameloblastic carcinoma			0 (0)	3 (7.3)	3 (7.3)
Total			2 (4.9)	39 (95.1)	41 (100)

* Fisher´s exact test

## Discussion

Oropharyngeal cancers were found to occur more in males than in females at a ratio of 2.7 to 1 with the mean age being 61years (Table 1). This is similar to what has been reported by authors from around the world where oropharyngeal cancers are said to be more than twice more likely to occur in males than in females and the mean age ranged from 62-63 years [4-6, 9]. This study found the overall prevalence of HPV in patients with oropharyngeal carcinoma (OPC) to be 17.1%. With the prevalence being higher among females than among the males at 27.3% to 13.3% (Table 2). This overall prevalence is similar to the 18.2% reported by researchers in Brazil [9] in a systematic review of 42 published articles on HPV in oropharyngeal cancer. They however did not elaborate on the sex specific prevalence. Another study by Castellsague *et al*. [10] found a higher prevalence of 22% among patients with oropharyngeal carcinomas.

There are few studies from Africa, and Nigeria in particular on the role of Human papilloma virus in OPC. In a retrospective study in the Central African Republic [11] where 135 head and neck cancer specimens from 2009-2017 were reviewed, the prevalence of HPV in head and neck cancer was found to be 0.74%. This low value was attributed to their storage protocol and the fact that their specimens were only from the oral cavity and the larynx. On the other hand, in a retrospective study carried out across 4 Nigerian teaching hospitals, where the head and neck cancer specimens from 1990 to 2011 were reviewed, no HPV positive OPC was detected. This the authors postulated to be likely due to their poor tissue processing. The high HPV positivity rate in this study could be attributed to the fact that our study was a descriptive cross sectional study hence the effect of storage that most of the retrospective studies from Africa encountered was not a factor in our study. This shows that significant differences in HPV+ rates for OPC from one study to the other could be because of the composition of the study population, distribution of anatomic sites considered as part of the oropharynx and the biomarkers used for calling a tumor HPV positive [12].

This study found a higher prevalence of HPV in females than in males (Table 2). This is in contrast to studies carried out recently in the United States of America and New Zealand [13, 14] where HPV was found to be more prevalent in males. It was said that the underlying causes of the observed gender differences in oropharyngeal cancer incidence was largely unknown [13]. However, the study carried out by D´Souza *et al*. [15] explained that the higher rates of HPV-related oropharyngeal cancers observed in men compared to women in the US was explained both by higher oral HPV acquisition rates, as well as slower clearance once infected as significant interactions were detected between gender, oral sexual behavior, and risk of incident oral HPV infection. This may also explain our own gender differences as none of the patients recruited for this study admitted to having oral sexual behavior. The age groups in this study except the 31-40 years age group were affected (Table 2). The 51-60 years as well as the greater than 70 years age groups had the highest prevalence. This showed that HPV associated oropharyngeal carcinomas (OPC) though occurred below 40 years of age, cuts across all age groups. This is similar to the findings by Mistro *et al*. [16] who also found HPV positive OPC was similar across all age groups. On the other hand, Elrefaey *et al*. [17] found the median age of HPV positive OPC to be 54 years.

In this study, among the patients with HPV positive OPC, HPV genotypes 16, 33, 35 and 52 were identified (Table 2). Genotype 16 was the commonest seen in 42.9% and the other 57.1% had other genotypes of which 28.6% of the patients had more than one genotype. Most studies have also identified HPV-16 as the most common genotype found in HPV positive OPC. In the study by Bratman *et al*. [18] HPV -16 was identified in 84% of cases and the other 16% was accounted for by HPV 33, 35 and 56. Goodman *et al*. [19] also identified genotypes 18, 31, 33, 35, 39, 45 and 52 as other genotypes associated with OPC. The importance of this information is that the presently available HPV vaccines are the bivalent (HPV 16, 18), the quadrivalent (HPV 6, 11, 16, 18) and the 9-valent (HPV 6, 11, 16, 18, 31, 33, 45, 52, and 58.) vaccines [20]. With more than 50% of cases of HPV positive OPC not caused by HPV-16 genotype in our environment, only the 9-valent prophylactic vaccine would be effective in majority of our patients. All age groups had one or two genotypes identified with the genotypes identified in the males being HPV 16, 33 and 52 while in the females´ HPV 16, 33 and 35 (Table 2). Most of the available studies did not particularly specify the gender distribution of the HPV genotypes in oropharyngeal cancer the emphasis is usually on HPV associated with cervical cancers. Most of the patients at presentation had palpable lymphadenopathy (Table 3); this is in keeping with findings from multiple studies [17, 21] that have shown that HPV-positive tumors are more likely to present with early tumor and higher nodal stages.

Our study also found that there was no significant association between smoking of tobacco or alcohol use and the presence of HPV (Table 4) This is similar to the findings by other authors who documented that HPV positive OPC are usually seen in younger adults with little or no smoking or drinking history [3, 20]. This study found 7 histological types of oropharyngeal cancers with oropharyngeal squamous cell carcinoma being the commonest at 75.6% followed by ameloblastic carcinoma seen in 7.3% of patients (Table 5). This finding is similar to what was documented in Maiduguri, Nigeria [22] where 73.3% of the oropharyngeal cancers were squamous cell carcinoma though the second commonest was Non-Hodgkin´s lymphoma. The female patients studied presented with only squamous cell carcinoma, extranodal Non-Hodgkin´s lymphoma and ameloblastic carcinoma while the seven different histological types were seen in the male patients. In this study, only patients with squamous cell carcinoma and ameloblastic carcinoma had human papilloma virus (Table 5). The 57.1% HPV infection rate in Squamous cell carcinoma patients in this study is lower than 70% HPV rates in patients with similar cancer in the developed countries. The higher squamous cell carcinoma rate in developed countries has been attributed to the rise in HPV associated OPC in the developed countries [23].

This study found that patients with ameloblastic carcinoma were 33.3% more likely to have more than one HPV genotype than patients with squamous cell carcinoma (Table 6). Ameloblastic carcinoma is a cancer that combines the features of ameloblastoma and squamous cell carcinoma and it may appear de novo or originate from a pre-existing ameloblastoma [24]. The relationship between HPV and ameloblastoma has been proposed by authors in the past [25]. It is believed that HPV may gain access to intraosseous lesions via contact with the overlying oral mucosa, surgical manipulation of the surrounding structures prior to tumor development, and early onset exposure to the HPV during invagination of the enamel organ. Correnti *et al*. [25] in their studies were able to identify both high risk and low risk HPV genotypes in the samples of patients with ameloblatomas. Our finding of HPV in the patients with ameloblastic carcinoma lends credence to the theory that there is a relationship between HPV and ameloblastoma.

Out of the 41 patients seen with oropharyngeal cancers only 4.9% were also HIV positive and HPV was not detected in any of the HIV positive patients (Table 6). HIV-infected individuals are said to be at increased risk of developing oropharyngeal cancers compared with the general population and HPV-positive OPC are also increasingly reported to be a significant cause of morbidity and mortality for HIV-infected individuals [26]. However, the proportion of HPV-positive oropharyngeal tumors in HIV-infected individuals is unknown [26]. The fact that we did not have many patients with HIV in the study population may have contributed to the finding of no significant association between HPV and HIV in this study. Kaposi´s sarcoma is the most common neoplasm associated with acquired immunodeficiency syndrome while Non-Hodgkin´s Lymphoma represents the second most common malignancy after Kaposi sarcoma in HIV positive patients with HIV-associated NHLs being extra nodal having a predilection for sites in the head and neck region in 50-60% of cases [27].

**Limitations:** this study has a few limitations, which could be taken into consideration in a larger study. They include the small sample size resulting from the major challenge of getting enough cases within the limited study period. Control subjects were not sampled to determine prevalent HPV genotypes in subjects without OPC and the low number of subjects with HIV to ascertain the roll of HIV in OPC in a Nigeria population.

## Conclusion

High-risk human papilloma virus genotypes 16, 33, 35 and 52 are associated with oropharyngeal carcinoma in adult Nigerians but were not found in HIV patients. This finding provides a strong evidence for the use of the 9-valent prophylactic vaccine for the prevention of oropharyngeal cancer in Nigeria. Public awareness and HPV prevention strategies should reduce significantly the incidence of oropharyngeal carcinomas in our environment.

### What is known about this topic

- Oropharyngeal carcinomas have essentially been a disease of the elderly;
- Incidence of HPV associated oropharyngeal carcinomas is on the rise world over but data from Nigeria obtained from retrospective studies have shown little or no HPV in oropharyngeal carcinomas in Nigeria.

### What this study adds

- Our study contributes to reinforcing the knowledge of HPV and oral carcinomas.
- High-risk human papilloma virus genotypes 16, 33, 35 and 52 are associated with oropharyngeal carcinoma in adult Nigerians with some individuals having more than one genotype; HPV associated oropharyngeal carcinomas cuts across all adult age groups;
- Our study provides additional evidence for the need to introduce the HPV vaccine in the immunization program.
